# HIV-1 APOBEC-context Mutations Detected by Next-Generation Sequencing in Plasma: Implications for Drug Resistance Interpretation

**DOI:** 10.1093/infdis/jiag277

**Published:** 2026-06-04

**Authors:** Marta Illescas-López, Paloma Muñoz Báez, Adolfo de Salazar, Rafael Delgado, Asunción Iborra, Juan Carlos Galán, Raquel Carracedo, Mayra Sigcha, Carla López-Causape, Ana Fuentes, Antonio Aguilera, Cristina Marco-Sánchez, Federico García, CoRIS, Santiago Moreno, Inma Jarrín, David Dalmau, M Luisa Navarro, Federico Garcia, Eva Poveda, Jose Antonio Iribarren, Félix Gutiérrez, Francesc Vidal, Juan Berenguer, Juan González, Inma Jarrín, Cristina Moreno, Marta Rava, Rebeca Izquierdo, Cristina Marco-Sánchez, Teresa Gómez García, José Luis Jiménez, Sergio Reus, Irene Portilla, Esperanza Merino, Gema García, José Sánchez-Payá, Juan Carlos Rodríguez, Livia Giner, Joaquín Portilla, Vicente Boix, Diego Torrus, Julia Portilla-Tamarit, Héctor Pinargote, María Remedios Alemán, Nereyda Tosco-García, Ana López Lirola, Dácil García, Felicitas Díaz-Flores, María del Mar Alonso, Ricardo Pelazas, María Inmaculada Hernández, Lucia Romero-Acevedo, Abraham Bethencourt-Padilla, Daniel Rodríguez-Díaz, Ana María Godoy-Reyes, Víctor Asensi, Rebeca Cabo-Magadan, Lorena Fernández, Javier Díaz-Arias, Federico Pulido, Rafael Rubio, M Asunción Hernando, Otilia Bisbal, David Rial-Crestelo, María de Lagarde, Laura Bermejo, Mireia Santacreu, Juan Martín Torres, Belén Sánchez-López, José Antonio Iribarren, Xabier Kortajarena, Claudia Nevado Pavón, Xabier Camino, Miguel Ángel Goenaga, M Jesús Bustinduy, Harkaitz Azkune, Maialen Ibarguren, Ignacio Álvarez-Rodriguez, Leire Gil-Alonso, Francisco Carmona-Torre, Ana Bayona Carlos, Maialen Lekuona Sanz, Leire Balerdi Sarasola, Ander Goyache Moreno, Félix Gutiérrez, Catalina Robledano, Mar Masiá, Sergio Padilla, Rafael Pascual, Marta Fernández, Antonio Galiana, José Alberto García, Xavier Barber, Javier García Abellán, Guillermo Telenti, Ángela Botella, Paula Mascarell, Lidia García-Sánchez, Nuria Ena, Leandro López, Jennifer Vallejo, Nieves Gonzalo-Jiménez, Montserrat Ruiz, Christian Ledesma, Santiago López, María Espinosa-Pérez, Ana Quiles, María del Mar Alcalde-Encinas, José García-García, Rosario Hernández-Ros, José Carlos Escribano, Marouane Menchi- Elanci, María del Mar García Navarro, Melissa Bello Pérez, Roberto Muga, Daniel Fuster, Cristina Diez, Isabel Gutiérrez, Juan Berenguer, Margarita Ramírez, Teresa Aldamiz-Echevarría, Francisco Tejerina, Leire Pérez-Latorre, Chiara Fanciulli, Saray Corral, Joaquim Peraire, Anna Rull, Anna Martí, Consuelo Viladés, Beatriz Villar, Lluïsa Guillem, Silvia Chafino, Marina Flores, Francesc Vidal, Marta Montero-Alonso, María Tasias-Pitarch, Eva Calabuig- Muñoz, Miguel Salavert-Lletí, Juan Fernández-Navarro, Rosa Blanes-Hernández, Jennifer Sánchez-Guevara, Juan González-García, José Ignacio Bernardino, Ana Delgado-Hierro, José Ramón Arribas, Víctor Arribas, Juan Miguel Castro, Luis Escosa, Iker Falces, Pedro Herranz-Pinto, Alicia González-Baeza, María Luz Martín-Carbonero, Rafael Micán, Rocío Montejano, María Luisa Montes, Luis Ramos-Ruperto, Berta Rodés Soldevila, Talia Sainz, Elena Sendagorta, Carmen Busca, Joanna Cano-Smith, Rosa de Miguel, María del Mar Arcos-Rueda, Alejandro de Gea-Grela, Nerea Iniesta-Arandia, Alejandro Díez-Vidal, María Jesús Roldán-Cabrales, Carlos Oñoro-López, Jara Llenas García, Laura Lucía Checa Daimiel, José Ramón Blanco, Laura Pérez-Martínez, Valvanera Ibarra, Luis Metola, Mercedes Sanz, Estrella Melús, Rosa Martínez-Álvarez, Desiré Gil-Pérez, Valentina Novarese, Ruth Caballero-Asensio, María Aranzazu Caudevilla-Martínez, María Espuelas-Monge, David Dalmau, Marina Martinez, Angels Jaén, Mireia Cairó, Javier Martinez-Lacasa, Roser Font, Laura Gisbert, María Rivero, Carlos Ibero-Esparza, Estela Moreno García, Fernando Baigorria Feltrin, Ana Miqueleiz Zapatero, Patricia Moreira Escriche, Andrés Enrique Blanco di Matteo, Victoria Duro Suárez, Gemma Navarro, Sonia Calzado Isbert, Marta Navarro Vilasaro, Juan Francisco Delgado, Germa Julia Agulló, Ignacio de los Santos, Alejandro de los Santos, Lucio García-Fraile, Enrique Martín-Gayo, Ildefonso Sánchez- Cerrillo, Ángela Gutiérrez, Carmen Sáez, Ana Barrios Blandino, Azucena Bautista, Marianela Ciudad, María Aguilera García, Violeta Sampériz Rubio, Javier Pérez Serrano, Isabel Belmonte Martín de Santa Olalla, Santiago Moreno, Santos del Campo, José Luis Casado, Fernando Dronda, Ana Moreno, María Jesús Pérez-Elías, Sergio Serrano-Villar, María Jesús Vivancos-Gallego, Javier Martínez-Sanz, Alejandro Vallejo Tiller, Matilde Sánchez-Conde, José Antonio Pérez-Molina, José Manuel Hermida, Erick De La Torre Tarazona, Elena Moreno del Olmo, Laura Martín Pedraza, Claudio Díaz García, Jorge Díaz Álvarez, Alejandro García-García, Raquel Ron-González, Sergio Calderón Vicente, Roser Navarro Soler, Sara Saiz-Baggetto, Ana del Amo-de Palacios, Laura Luna, Miguel Antón Ámez Segovia, Sara Martín Colmenarejo, Cristina Chica, Enrique Bernal, María Dolores Hernández, Antonia Alcaraz, Joaquín Bravo, Ángeles Muñoz Pérez, Cristina Tomás Jimenez, Salvador Valero Cifuentes, Eva García-Villalba, Román González Hipólito, Elena Guijarro-Westermeyer, Rodrigo Martínez-Rodríguez, José Miguel Gómez Verdú, Federico García, Clara Martínez, Maite Laperal, Leopoldo Muñoz Medina, Marta Álvarez-Estevez, Natalia Chueca-Porcuna, David Vinuesa-García, Adolfo de Salazar-González, Ana Fuentes-López, Emilio Guirao, Andrés Ruiz-Sancho, Francisco Anguita, Naya Faro, Lucia Chaves, Marta Illescas, Paloma Muñoz-Baez, Lucía Pérez, Ana Alberola Romano, Alberto Vazquez Blanquiño, Lucía Guillén-Zafra, Javier Martínez de Victoria-Carazo, Jorge Del Romero-Guerrero, Montserrat Raposo, Teresa Puerta-López, Mar Vera, Juan Ballesteros, Begoña Baza, Laura Dans Villán, Ruben Linares Navarro, Ines Armenteros Yeguas, Eva Orviz-García, Santiago Fernández Castelao, Antonio Antela, Sandra Daponte, Elena Losada, Melchor Riera, Antonio Vanrell, María Peñaranda, Mª Angels Ribas, Antoni A Campins, Mercedes Garcia-Gasalla, Francisco J Fanjul, Javier Murillas-Angoiti, Luisa Martin-Pena, Francisca Artigues, Sophia Pinecki, Adrián Ferre, Jesús Santos, María López-Jódar, Cristina Gómez-Ayerbe, Isabel Viciana, Rosario Palacios, Luis Fernando López-Cortés, Silvia Llaves-Flores, Inmaculada Rivas Jeremías, Nuria Espinosa, Cristina Roca-Oporto, Marta Herreros-Romero, César Sotomayor de la Piedra, Abraham Saborido Alconchel, Manuel Francisco Liroa, Jesús Fernández Plaza, Juan Manuel Tiraboschi, Camila Soledad Piatti, Arkaitz Imaz, María Saumoy, Analuz Fernandez, Jaime Vega Costa, Daniel Medina Gamito, Adrián Curran, Vicenç Falcó, Jordi Navarro, Joaquin Burgos, Paula Suanzes, Jorge García, Vicente Descalzo, Patricia Álvarez, Bibiana Planas, Marta Sanchíz, Arnau Monforte, Julián Olalla, Javier Pérez, Alfonso del Arco, Javier de la Torre, Francisca Ruiz, Onofre Juan Martínez, Lorena Martinez, Francisco Jesús Vera, Josefina García, Begoña Alcaraz, Sergio Guillén Martínez, Patricia Carles García, Álvaro Mena de Cea, Brais Castelo, Pilar Vázquez-Rodríguez, Soledad López-Calvo, Verónica Pazos López, Sofía Ibarra-Ugarte, Guillermo García, Josu Mirena, Oscar Luis Ferrero, Mireia de la Peña, Miriam López, Iñigo López-Azkarreta, Itxaso Lombide-Aguirre, Víctor Polo, Joana de Miguel, Beatriz Ruiz-Estevez, Maite Ganchegui-Aguirre, María Jesús Barberá-Gracia, Mónica Vélez Tobarias, Isabel Izuzquiza-Avanzini, Jimena Varona Pérez, María Iriarte-Ibarrarán, Carlos Galera, Marian Fernández, Helena Albendin, Antonia Castillo, Asunción Iborra, Antonio Moreno, María Angustias Merlos, Almudena Ortuño, Concha Amador, Francisco Pasquau, Concepción Gil, José Tomás Algado, Inés Suarez-García, Eduardo Malmierca, Patricia González-Ruano, Mª Pilar Ruiz, Luz Balsalobre, Ángela Somodevilla, Rebeca Fuerte Martínez, María de la Villa López, Mohamed Omar, Carmen Herrero, Miguel Alberto de Zárraga, Desirée Pérez, Carlos Tarrazo, Juan Valdes Becares, Vicente Estrada, Nieves Sanz, Noemí Cabello-Clotet, María José Núñez, Ana Muñoz, Juncal Pérez-Somarriba, Reynaldo Homen, Rafael Rubio-Martín, Susana Olmedo, Julia Barrado, Miguel Górgolas, Alfonso Cabello, Cristina Algar Arévalo, Beatriz Álvarez, Laura Prieto, Aws Al-Hayani, Irene Carrillo, Marta López de las Heras, José Sanz, Cristina Hernández-Gutiérrez, María Novella-Mena, María José Galindo, Sandra Pérez Gómez, Ana Ferrer, Anaïs Corma Gómez, Antonio Rivero-Román, Laura Ruiz-Torres, Antonio Rivero-Juárez, Pedro López-López, Mario Frias-Casas, Ángela Camacho, Ignacio Pérez, Diana Corona, Javier Manuel Caballero, Marina Gallo Marín, María Casares, Lucía Ríos-Muñoz, Claudia Ferreira-Tata, María Carrasquilla, Rafael Rodríguez-Rosado Martinez-Echevarría, Juan Macías Sánchez, Pilar Rincón, Luis Miguel Real, Jésica Martín-Carmona, Eva Poveda, Alexandre Pérez, Luis Morano, Celia Miralles, Antonio Ocampo, Jacobo Alonso, Inés Martínez, Aida López-López, Carlos Dueñas, Pablo Telleria, Laura Rodríguez-Fernández, Marta de la Fuente López, Marina García de Vicuña, Sara Gómez García, Alberto Rodríguez-Iglesias, Roberto Güerri Fernández, Angela Requena Company, Juan Du, Agustin Marcos Blanco, Itziar Arrieta Aldea, Esperanza Cañas-Ruano, Cecilia Canepa, Natalia García Giralt, Jade Soldado, Irene Carbonell, María José Fernández Quiroga, Carlos de Andrés David, Karenina Antelo Cuéllar, Inmaculada Poquet Catalá, María Josefa de la Asunción Villaverde, Laira Navarro Peiró, Jorge Sánchez Villegas, Virginia Palomo Jiménez, Aurora Alemán Rodríguez, Lola Cubero Aranda, M Carmen Fariñas Álvarez, Claudia González Rico, Noelia Ruiz Alonso, Carlos Armiñanzas Castillo, Francisco Arnaiz de las Revillas Almajano, Manuel Gutiérrez Cuadra, Raúl Parra Fariñas, Paula Runza Buznego, Aitziber Illaro Uranga, José Luis Blanco Arévalo, Pilar Callau Cabrera, Josep Mallolas Masferrer, José Alcamí Pertejo, Sonsoles Sánchez-Palomino, Núria Climent, Ana González-Cordón, Montse Laguno Centeno, María Martínez-Rebollar, Juan Ambrosioni, Berta Torres, Lorena de la Mora, Alexy Inciarte, Esteban Martínez, José María Miró, Abiu Sempere, Julia Calvo, Iván Chivite, David García, Alberto Foncillas, Daniela Malano, Montse Plana, Roger Llobet, Estela Solbes, Octavi Roman, Rona Sagarra, Vanesa Guilera, Gemma Olmeda, Paula Arreba, María José Rodríguez, Emma Fernández, Ana Rodríguez, Alba ortega, Sergi Anguera, Raquel Aguiló, Laura Novell, Luis Miguel Bedoya Del Olmo, Manuela Beltran Vicente, Mercedes Bermejo Herrero, Maria Esther Calonge Errejon, Laura Capa Muñoz, Maria Teresa Coiras Lopez, Francisco Diez Fuertes, Javier Garcia Perez, Nuria Gonzalez Fernandez, Elena Mateos De Las Moreras, Maria Teresa Perez Olmeda, Victor Sanchez Merino, Maria Eloisa Yuste Herranz, Carmen Elena Gomez Rodriguez, Juan Francisco Garcia Arriaza, Laura Sin Diaz, Beatriz Perdiguero de la Torre, Laura Marcos Villar, Patricia Perez Ramirez, Enrique Alvarez Coruña, David Astorgano Lopez, Cristina Sanchez Corzo

**Affiliations:** Hospital Universitario de San Cecilio de Granada, Instituto de Investigación Ibs.Granada, Ciber de Enfermedades Infecciosas Ciberinfec, Madrid, Spain; Hospital Universitario de San Cecilio de Granada, Instituto de Investigación Ibs.Granada, Ciber de Enfermedades Infecciosas Ciberinfec, Madrid, Spain; Hospital Universitario de San Cecilio de Granada, Instituto de Investigación Ibs.Granada, Ciber de Enfermedades Infecciosas Ciberinfec, Madrid, Spain; Hospital Universitario 12 de Octubre, Madrid, Ciber de Enfermedades Infecciosas Ciberinfec, Madrid, Spain; Hospital Universitario Virgen de la Arrixaca, Murcia, Spain; Hospital Ramón y Cajal, Madrid, Ciber de Epidemiología y Salud Pública, Madrid, Spain; Departamento de Microbiologia, Hospital Clínico Universitario de Santiago de Compostela, Santiago de Compostela, Spain; Hospital Universitario 12 de Octubre, Madrid, Ciber de Enfermedades Infecciosas Ciberinfec, Madrid, Spain; Departamento de Microbiologia, Hospital Universitario Son Espases, Palma de Mallorca; Ciber de Enfermedades Infecciosas Ciberinfec, Madrid, Spain; Hospital Universitario de San Cecilio de Granada, Instituto de Investigación Ibs.Granada, Ciber de Enfermedades Infecciosas Ciberinfec, Madrid, Spain; Departamento de Microbiologia, Hospital Clínico Universitario de Santiago de Compostela, Santiago de Compostela, Spain; Centro Nacional de Epidemiología, Instituto de Salud Carlos III, Madrid, Spain; Hospital Universitario de San Cecilio de Granada, Instituto de Investigación Ibs.Granada, Ciber de Enfermedades Infecciosas Ciberinfec, Madrid, Spain

**Keywords:** HIV-1, resistance, APOBEC, NGS-Genotyping, integrase

## Abstract

**Background:**

APOBEC3-mediated cytidine deamination produces G→A substitutions in HIV genomes, described in proviral DNA but detectable in plasma HIV-1 RNA using NGS. APOBEC-signature mutations reflect G→A enrichment substitutions, whereas APOBEC-context mutations are substitutions at drug resistance-associated positions compatible with APOBEC editing. We evaluated theirprevalence and impact on resistance interpretation.

**Methods:**

We retrospectively analysed plasma HIV-1 NGS-sequences from antiretroviral-naive individuals in 2022–2023. Sequencing was performed in participating laboratories, and FASTQ centrally reanalyzed using DeepChek. APOBEC-signature mutations, APOBEC-context drug resistance mutations (DRMs), and stop codons were assessed in protease, reverse transcriptase, and integrase at 1%, 3%, and 5% thesholds.

**Results:**

Among 290 individuals, 268 had complete NGS. At 1%, APOBEC-signature mutations were detected in 159 individuals and APOBEC-context DRMs in 64 (22%). Both patterns coexisted in 52 (18%), decreasing to 17 (6%) and 8 (3%) at 3% and 5% thresholds. Certain mutations, including M184I, G190E, G140S, and R263K, remained detectable at higher thresholds. R263K co-occurred with multiple APOBEC-signature mutations and stop codons indicating extensive APOBEC-mediated editing.

**Conclusions:**

APOBEC-associated mutations are detectable in plasma HIV-1 RNA and may affect resistance interpretation. Conservative frequency thresholds (5%) APOBEC-signatures evaluation may reduce misclassification of APOBEC-induced variants as clinically relevant resistance.

## Teaser

APOBEC-associated mutations are detectable in plasma HIV-1 by next-generation sequencing and may confound resistance interpretation. Applying conservative frequency thresholds and assessing APOBEC signatures can help distinguish clinically relevant variants from APOBEC-induced changes.

## Graphical Abstract

**Graphical Abstract:**
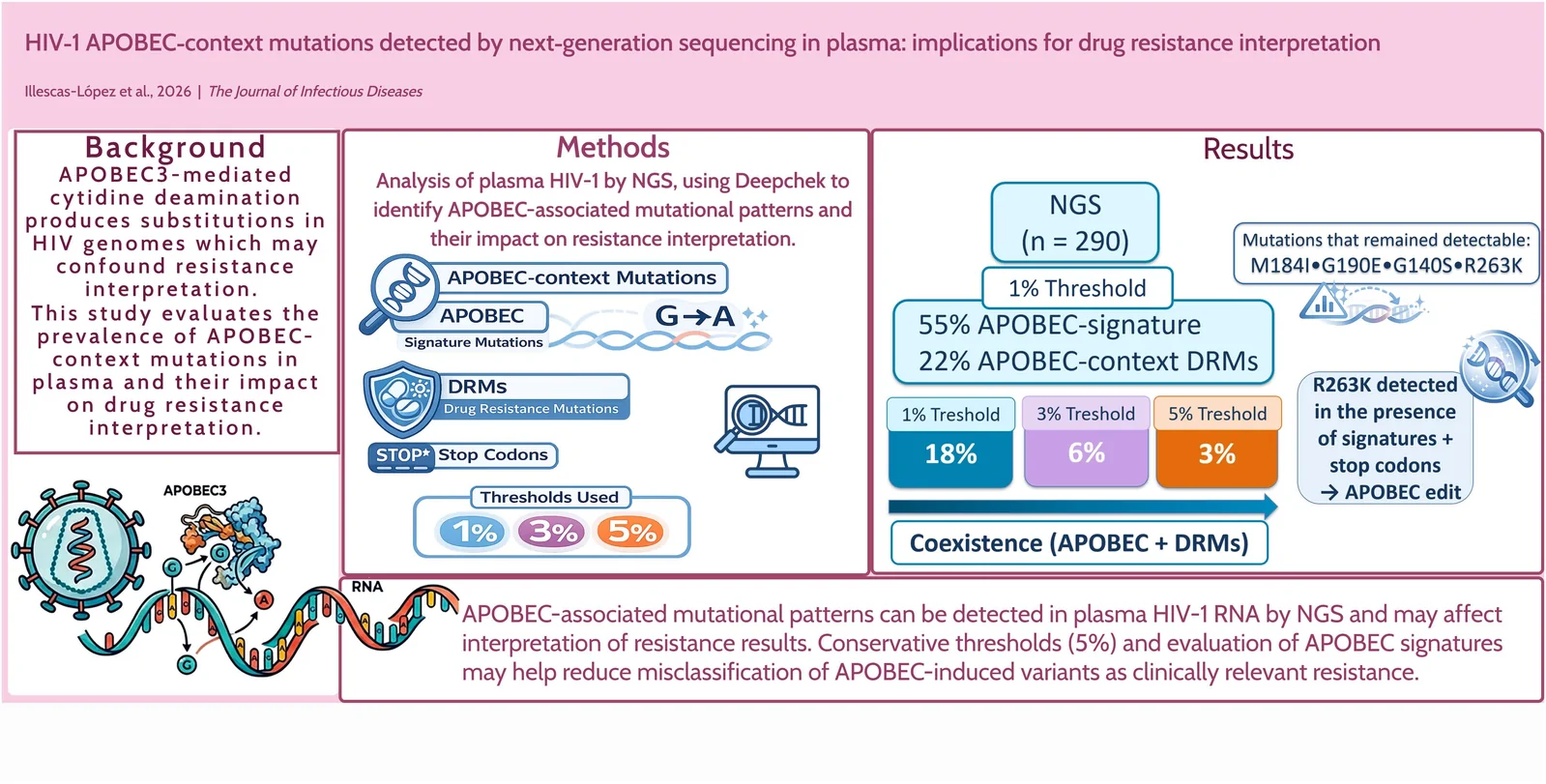

The apolipoprotein B mRNA editing enzyme catalytic polypeptide-like 3 (APOBEC3) family constitutes a key component of the intrinsic antiviral immune response against HIV-1. APOBEC3 proteins, particularly APOBEC3G and APOBEC3F, restrict viral replication by deaminating cytidine residues during reverse transcription, resulting in guanosine-to-adenosine (G→A) substitutions in the viral DNA genome [1, 2]. While extensive APOBEC-mediated editing frequently leads to lethal hypermutation and defective proviruses, lower levels of APOBEC activity can leave characteristic G→A mutational footprints in viral sequences, particularly within well-described APOBEC target motifs. These patterns can be recognized in sequence datasets and are commonly used to infer APOBEC-mediated editing in HIV genomic analyses [3].

Although APOBEC-driven mutations are more commonly described in cell-associated HIV-1 DNA [4, 5], they can also be detected in plasma HIV-1 RNA, indicating that APOBEC-associated mutational patterns may be present in circulating virus despite incomplete elimination of viral genomes by APOBEC activity [6, 7]. In plasma, these mutations are often present at low frequencies and may escape detection by conventional Sanger sequencing. However, the increasing implementation of next-generation sequencing (NGS) for resistance testing has markedly improved sensitivity for minority variants, thereby increasing the likelihood of identifying APOBEC-related mutations in circulating virus.

This has important implications for the interpretation of HIV drug resistance. Several amino acid substitutions at positions associated with antiretroviral resistance can arise as a direct consequence of APOBEC-mediated G→A editing rather than antiretroviral selective pressure [3, 8]. The Stanford HIV Drug Resistance Database has formalized this distinction by defining APOBEC signature mutations, which reflect an overall enrichment of APOBEC-related editi-g, and APOBEC-context mutations, corresponding to specific substitutions at drug resistance-associated positions consistent with known APOBEC target motifs [9]. APOBEC-signature mutations refer to a broader pattern of G→A substitutions occurring in characteristic APOBEC target sequence contexts (typically 5′-GG→AG or 5′-GA→AA), reflecting the cumulative activity of APOBEC enzymes across the viral genome [10–12]. In contrast, APOBEC-context mutations correspond to specific nucleotide substitutions at drug resistance-associated positions that occur within known APOBEC target motifs and may therefore arise as a direct consequence of APOBEC editing [13–15]. The identification of multiple APOBEC-signature mutations within the same sequence supports the presence of APOBEC-mediated editing and may help interpreting whether APOBEC-context mutations are likely to reflect APOBEC activity rather than antiretroviral selective pressure [16, 17]. Importantly, APOBEC-context mutations occurring in isolation, without accompanying APOBEC-signature mutations, may represent genuine resistance-associated variants [3]. In the setting of NGS-based plasma genotyping, failure to recognize these mutations may lead to misclassification of APOBEC-induced changes as clinically relevant resistance, particularly when APOBEC-context DRMs are detected in sequences that also display broader APOBEC-associated patterns, such as multiple APOBEC-signature mutations or stop codons consistent with hypermutation. In this study, we analyze APOBEC-context DRMs in plasma HIV-1 sequences generated by NGS, highlighting their prevalence and emphasizing the need for careful interpretation to avoid overestimation of antiretroviral resistance in clinical and surveillance settings.

## METHODS

We performed a retrospective analysis of plasma HIV-1 sequences from newly diagnosed individuals included in the CoRIS resistance work-package, with NGS data available between 2022 and 2023. CoRIS is an open, prospective cohort enrolling antiretroviral-naive people with HIV aged >13 years and followed at HIV units across Spain [18, 19]. All individuals were antiretroviral-naive at the time of sampling. A small number of additional individuals fulfilling the same eligibility criteria and managed under the same clinical and analytical workflow were also included. NGS-based resistance testing was performed in participating clinical microbiology laboratories as part of routine clinical care using Illumina sequencing platforms (MiSeq, n = 4; iSeq, n = 1, NextSeq, n = 1). Plasma samples were generally collected in K2-EDTA tubes and processed according to the standard procedures of each participating center: blood samples were collected in hospital settings and transported to a central reception area, from which they were distributed to the corresponding microbiology laboratories. Plasma was separated according to routine clinical workflows and stored at −80°C until processing. RNA was extracted from plasma samples typically obtained from 300–1000 µL of plasma, depending on local laboratory protocols (EZ1 Advanced XL (QIAGEN), n = 2; NucliSENS® easyMAG® (bioMérieux), n = 2; MagCore® HF16 Automated Nucleic Acid Extractor (RBC Bioscience Corp.), n = 1; TANBead® Nucleic Acid Extractor, n = 1) following the protocols implemented in each center. Pre-analytical timelines, including time from collection to processing, were not standardized across centers and reflect real-world clinical practice. DNase treatment or specific assessment of HIV DNA carryover was not routinely performed, in accordance with standard protocols provided by the manufacturers and commonly applied in clinical diagnostic laboratories. In general, samples processed for genotypic resistance testing correspond to individuals with plasma HIV-1 RNA levels above 500–1000 copies/mL, reflecting standard clinical practice across centers.

Amplification of the HIV-1 genomic regions of interest (protease, reverse transcriptase and integrase) was performed using commercially available NGS resistance genotyping kits: HIV-1 Solution v2, Arrow Diagnostics (https://www.arrowdiagnostics.it/en/news/view.php?id=5), n = 3; HIV-1 Protease/reverse transcriptase & Integrase Genotyping and Drug Resistance ABL Diagnostics (https://www.abldiagnostics.com/products/hiv-genotyping-drug-resistance/, n = 3), according to the protocols implemented at each center. These sequences were generated as part of routine clinical care and therefore reflect real-world laboratory practices across the participating sites. Raw sequencing data (FASTQ files) generated at the participating centers were subsequently transferred to the coordinating center for centralized analysis. All FASTQ files were reanalyzed in batch using DeepChek® [20]. Variant calling and sequence analysis were performed using uniform quality control criteria applied to all samples. These included a variant frequency cutoff of 1%, a minimum coverage of 100 reads at the nucleotide position of interest, and a minimum base quality score (Q-score) > 20. These filters were applied to minimize the contribution of low-confidence calls and sequencing background noise to mutation detection.

APOBEC-context DRMs and APOBEC-signature mutations were identified and evaluated according to established definitions provided in the Introduction. Both APOBEC-signature and -context mutations were identified according to the Stanford HIV Drug Resistance Database. For each mutation, relative prevalence was calculated, and sequence reliability was assessed using the quality index of precision (Q-score). In addition, the presence of stop codons was recorded as a marker of extensive APOBEC-mediated editing.

Within the CoRIS cohort, all participants provided written informed consent, and the study protocol was approved by the ethics committees of all participating centers. Artificial intelligence–based tools were used exclusively for language editing and clarity improvement; no AI tools were used for data analysis, data interpretation, or decision-making.

## RESULTS

A total of 290 people living with HIV were included in the analysis; among them, 268 had NGS data available for all 3 genomic regions (protease, reverse transcriptase, and integrase). Using a 1% frequency cutoff, APOBEC-signature mutations were detected in 159 individuals, and APOBEC-context DRMs were identified in 64 participants (22%). The coexistence of APOBEC-signature mutations and APOBEC-context DRMs was observed in 52 individuals (18%) using a 1% cutoff, decreasing to 17 (6%) and 8 (3%) when applying frequency thresholds of 3% and 5%, respectively. No relevant differences in the detection of APOBEC-associated patterns were observed across participating centers.

APOBEC-context DRMs were identified across all 3 genomic regions analyzed (protease, reverse transcriptase, and integrase), with marked differences in prevalence according to the frequency cutoff applied. At the 1% threshold, APOBEC-context mutations were most frequently observed in reverse transcriptase, particularly M184I and M230I, whereas fewer mutations were detected in protease and integrase. Increasing the threshold to 3% and 5% resulted in a marked reduction in the number of detected APOBEC-context mutations across all regions, consistent with the expected loss of low-frequency variants at more stringent cutoffs.

Several mutations observed at the 1% cutoff were no longer detected at higher thresholds, particularly in protease and reverse transcriptase. This effect reflects the general reduction in the detection of low-frequency variants when higher thresholds are applied in NGS analyses, which may also affect other types of sequence variants such as polymorphisms, DRMs, or stop codons. Notably, M184I and G190E in reverse transcriptase, as well as G140S and R263K in integrase, remained detectable at higher cutoffs, although with markedly lower frequencies (Table 1).

**Table 1.** APOBEC-context Drug Resistance Mutations Detected in Plasma HIV-1 by Next-Generation Sequencing at Different Frequency Cutoffs. APOBEC-context Drug Resistance Mutations (DRMs) Detected in the Protease (PR), Reverse Transcriptase (RT), and Integrase (IN) Regions Using Frequency Thresholds of 1%, 3%, and 5%. Numbers Indicate the Count of Individuals With Each Mutation. the Predicted Impact on Antiretroviral Drug Susceptibility is Reported According to the Stanford HIV Drug Resistance Database Interpretation

		1%-Cutoff	3%-Cutoff	5%-Cutoff	Impact
PR	D30N	3	2	1	…
	M46I	18	2	1	…
	G73S	3	…	…	…
RT	D67N	4	…	…	AZT(I)
	E138K	3	…	…	RPV(I)
	M184I	32	3	1	3TC/FTC (R); ABC (I)
	G190E	6	1	1	EFV, DOR, RPV (R); ETV(I)
	M230I	32	8	…	RPV (I)
IN	E138K	5	…	…	CAB(I)
	G140S	4	2	1	CAB (I)
	G140R	2	…	…	CAB(R)
	R263K	5	3	3	CAB(R); BIC, DTG (I)

According to the Stanford HIV Drug Resistance Database interpretation, the identified APOBEC-context mutations were associated with predicted reduced susceptibility or resistance to drugs from all major antiretroviral classes (Table 1). These included nucleos(t)ide reverse transcriptase inhibitors (eg, M184I affecting lamivudine/emtricitabine and abacavir), non-nucleoside reverse transcriptase inhibitors (eg, G190E and E138K impacting efavirenz, doravirine, rilpivirine, and etravirine), and integrase strand transfer inhibitors (eg, G140S/R and R263K affecting cabotegravir, with intermediate impact on bictegravir and dolutegravir). In contrast, APOBEC-context mutations detected in protease were not associated with a predicted impact on protease inhibitor susceptibility. These interpretations correspond to the resistance profiles assigned by the Stanford algorithm when such substitutions are considered individually at known drug resistance positions.

When restricting the analysis to variants detected at a ≥ 5% frequency threshold, distinct APOBEC-associated mutational patterns were observed in reverse transcriptase and integrase (Tables 2). In reverse transcriptase, sequences harboring APOBEC-context mutations M184I and G190E showed a limited number of accompanying APOBEC-signature mutations, with few additional APOBEC-consistent substitutions and a low prevalence of stop codons. In contrast, a markedly different pattern was observed in integrase sequences carrying the APOBEC-context mutation R263K. At the 5% cutoff, R263K was frequently associated with a high number of concomitant APOBEC-signature mutations, consistent with widespread APOBEC-mediated editing. Notably, sequences containing R263K also exhibited a high prevalence of stop codons, indicative of extensive G→A hypermutation and the likely presence of defective viral genomes. This enrichment of APOBEC-signature mutations and stop codons was substantially greater than that observed for APOBEC-context mutations in reverse transcriptase. This effect was also seen for M46I in the protease.

**Table 2.** APOBEC-Context Drug Resistance Mutations Detected at ≥5% Frequency and Associated APOBEC-signature Mutations Across Protease, Reverse Transcriptase, and Integrase

APOBEC-context (%)	Gene	APOBEC-Signature (PRO)	APOBEC-Signature (RT)	APOBEC-Signature (IN)	Subtype	Viral Load
D30N (14%)	PRO	none	none	E152K; E157K	B	1205
M46I (8%)	PRO	G40E; G40R; W42*; G48R; G51R; G52S; G86E; G86R	G18S; G45R; G51R; E53K; W71*; R72K; E79K; G93E; G152E; W153*; D186N; W229*; E233K; W239*; W252*; W266*; G285E; E302K; E305K	E48K; D64N; E69K; E87K; G106E; W131*; W132*; G140E; G149R; E157K; R166K; D167N; G192R; E198K; R199K; G237R; W243*; E246K; G247R; R262K	B	53 375
M184I (6%)	RT	W24*; G112S; D185N	none	E152K	B	564
G190E (12%)	RT	W42*	R72K; E89K	G47E; E48K; E157K; R224Q	B	531
G140S (13%)	IN	none	G384E	none	B	8500
R263K (7%)	IN	D29N; W42*; G48R; G49R; G51R; G52S	G15R; G18R; G18S; M41I; G45R; G51R; E53K; W71*; R72K; E79K; G99R; G152E; W153*; E233K; W252*; W266*; G285E; E305K	E48K; D55N; D64N; E69K; E85K; E87K; G106E; R107K; W131*; W132*; E152K; E157K; R166K; D167N; E170K; M178I; G192R; E198K; R199K; R228K; W235*; W243*; E246K; R262K	A1	506 251
R263K (7%)	IN	D29N; W42*; G48R; G51R; G52S	G15R; G18R; G18S; M41I G45R; G51R; E53K; W71*; G152E; W153*; W229*; W239*; E302K	E48K; E69K; G70R; G82S; E85K; E87K; E92K; G106E; R107K; G149E; E157K; R166K; E170K; G192R; E198K; R199K; W235*; W243*; E246K; G247R; D256N; R262K; G272R	B	7 611 086
R263K (8%)	IN	W42*; G48R; G51R; G52S; G86R	G15R; G18R; G18S; M41I; G45R; G51R; E53K; W71*; R72K; W88*; G93E; D110N; G152E; W153*; W229*; E233K; W252*; G262R; W266*; E305K	D55N; D64N; E69K; E87K; E92K; R107K; W131*; G149R; E152K; R166K; D167N; E170K; E198K; R199K; W235*; W243*; E246K; G247R; R262K; G272R	…	79 823

APOBEC-context drug resistance mutations (DRMs) and associated APOBEC-signature mutations detected at a≥5% frequency threshold by next-generation sequencing (NGS) in protease (PR), reverse transcriptase (RT), and integrase (IN). APOBEC-context mutations are defined according to the Stanford HIV Drug Resistance Database and are shown with their corresponding variant frequency (%). APOBEC-signature mutations represent additional substitutions consistent with APOBEC-mediated G→A editing and are presented by genomic region. APOBEC-signature mutations presented in bold are those >5% prevalence. The presence of APOBEC-signature mutations in regions different from the APOBEC-context mutation indicates cross-regional APOBEC-associated patterns within the same sample. The asterisk (*) denotes the presence of a stop codon.

## DISCUSSION

Although APOBEC-mediated mutagenesis has classically been considered a confounding factor primarily affecting proviral HIV-1 DNA, our findings demonstrate that APOBEC-context DRMs and extensive APOBEC-signature patterns can also be detected in plasma HIV-1 RNA when NGS is applied. Previous studies, including work by Gifford et al [21]. and the Stanford group [9, 22], have already highlighted the importance of APOBEC-mediated editing in the interpretation of HIV resistance mutations. Our findings extend these observations by showing that APOBEC-associated patterns may also be detected in NGS-based plasma HIV-1 RNA sequences obtained in routine clinical practice from newly diagnosed individuals. The increased sensitivity of NGS for low-frequency variants makes the identification of APOBEC-related mutations in plasma more likely, even in newly diagnosed individuals. This is particularly relevant for resistance interpretation, as several APOBEC-context mutations affect positions associated with clinically meaningful antiretroviral resistance.

Our findings also suggest that the choice of variant frequency threshold may substantially influence the interpretation of NGS-based resistance testing. While lower thresholds such as 1% may increase analytical sensitivity, and may be useful for exploratory analyses and for detecting the full spectrum of low-frequency associated variants, they also increase the likelihood of detecting low-frequency variants arising from APOBEC-mediated editing or other background processes. In this context, our results support, as other studies [23, 24] the use of more conservative thresholds—around 5%—for routine clinical resistance interpretation, which may reduce the risk of misclassifying APOBEC-associated variants as clinically relevant resistance mutations.

Our data support the need to actively screen for APOBEC signatures when interpreting NGS-based plasma genotypes, in order to avoid misclassification of APOBEC-induced variants as resistance mutations and the consequent risk of overestimating resistance, especially for integrase strand transfer inhibitors. For this purpose, the Stanford HIV Drug Resistance Database may help guide interpretation, although it does not currently incorporate variant frequency. This concern is especially relevant because some APOBEC-context integrase mutations, such as R263K, were frequently observed together with multiple APOBEC-signature mutations and stop codons, suggesting that they may arise within heavily APOBEC-edited and potentially defective viral genomes rather than representing clinically meaningful resistance variants. These findings should be interpreted in the context of a newly diagnosed, antiretroviral-naive population, in whom APOBEC-context mutations are unlikely to reflect prior treatment-driven selection. In other clinical settings, particularly in treatment-experienced individuals, APOBEC-context mutations may coexist with genuine drug-selected resistance mutations and therefore require even more careful contextual interpretation.

A major limitation of this study is that it relied on short-read NGS data and did not incorporate long-read sequencing approaches, such as nanopore-based single-genome sequencing. Therefore, we cannot conclusively demonstrate linkage between APOBEC-context DRMs and accompanying APOBEC-signature mutations or stop codons within the same viral genome [25, 26]. Establishing linkage between APOBEC-context DRMs, accompanying APOBEC-signature mutations, and stop codons is important because it determines whether these changes occur within the same viral genome. If linked, such patterns would support the interpretation that the resistance-associated substitution is embedded within a heavily APOBEC-edited, and potentially defective, viral sequence [27]. In contrast, if unlinked, the clinical and biological interpretation may be different [7]. For this reason, long-read sequencing approaches will be important to define the genomic structure and relevance of APOBEC-associated variants detected in plasma. Future studies using long-read sequencing technologies will be necessary to confirm genomic linkage and to better delineate the structure of APOBEC-edited viral genomes detected in plasma.

An additional limitation relates to the potential contribution of HIV DNA to the analyzed sequences. Pre-analytical conditions, including time from sample collection to plasma separation and storage, were not standardized across participating centers and followed routine clinical workflows. This variability may influence the likelihood of cellular DNA carryover into plasma samples. In addition, RNA extraction was performed using automated commercial platforms without DNase treatment, which is not routinely included in manufacturer-recommended protocols for clinical resistance testing. As a result, a minor contribution of proviral HIV DNA cannot be completely excluded and may potentially contribute to the detection of APOBEC-associated mutational patterns. However, these conditions reflect real-world clinical practice and therefore support the generalizability of our findings to routine diagnostic settings.

Our analysis was specifically focused on APOBEC-context DRMs because these are the substitutions most likely to generate misleading resistance interpretations in routine NGS reports. Although other DRMs may also be detected in these sequences, our aim was not to provide a comprehensive resistance profile but to assess the subset of variants most directly related to APOBEC-mediated editing and its potential impact on clinical interpretation.

We also acknowledge that formal comparison with previously described computational approaches for detecting APOBEC-mediated hypermutation, such as the Hypermut tool available through the Los Alamos HIV Sequence Database [28], was not the primary objective of this study. Our approach instead focused on APOBEC-signature mutations, APOBEC-context mutations, and stop codons as complementary markers of putative APOBEC-mediated editing in NGS-derived plasma sequences. Future studies directly comparing these approaches may help refine the interpretation of APOBEC-associated patterns in plasma HIV-1 RNA.

Overall, our findings highlight that APOBEC-context mutations detected at clinically relevant resistance positions may arise in distinct biological contexts. While APOBEC-context mutations in reverse transcriptase can be detected as isolated events at higher frequencies; of special interest, in our study the integrase mutation R263K appears to preferentially occur within heavily APOBEC-edited viral genomes. Accordingly, APOBEC-context mutations identified by NGS should be interpreted cautiously, particularly when detected at low frequencies or in sequences showing multiple APOBEC-signature mutations and stop codons. Rather than indicating clinically meaningful resistance by themselves, such variants may in some cases reflect APOBEC-mediated editing within biologically compromised viral populations. Laboratories using NGS should therefore consider APOBEC-associated metrics when interpreting resistance results, especially in the setting of highly sensitive variant detection thresholds.

## References

[1] Malim MH . APOBEC proteins and intrinsic resistance to HIV-1 infection. Philos Trans R Soc B Biol Sci 2009; 364:675–87. 10.1098/rstb.2008.0185 PMC2660912 19038776

[2] Okada A, Iwatani Y. APOBEC3G-mediated G-to-A hypermutation of the HIV-1 genome: the missing link in antiviral molecular mechanisms. Front Microbiol 2016; 07(DEC):1–8. 10.3389/fmicb.2016.02027 PMC5165236 28066353

[3] Venkatesan S, Rosenthal R, Kanu N, et al Perspective: APOBEC mutagenesis in drug resistance and immune escape in HIV and cancer evolution. Ann Oncol 2018; 29:563–72. 10.1093/annonc/mdy003 PMC5888943 29324969

[4] Jern P, Russell RA, Pathak VK, Coffin JM. Likely role of APOBEC3G-mediated G-to-A mutations in HIV-1 evolution and drug resistance. PLoS Pathog 2009; 5:e1000367. 10.1371/journal.ppat.1000367 PMC2659435 19343218

[5] Armenia D, Gagliardini R, Alteri C, et al Temporal trend of drug-resistance and APOBEC editing in PBMC genotypic resistance tests from HIV-1 infected virologically suppressed individuals. J Clin Virol 2023; 168(July):105551. 10.1016/j.jcv.2023.105551 37573167

[6] Sato K, Izumi T, Misawa N, et al Remarkable lethal G-to-A mutations in vif- proficient HIV-1 provirus by individual APOBEC3 proteins in humanized mice. J Virol 2010; 84:9546–56. 10.1128/JVI.00823-10 PMC2937654 20610708

[7] Noguera-Julian M, Cozzi-Lepri A, Di Giallonardo F, et al Contribution of APOBEC3G/F activity to the development of low-abundance drug-resistant human immunodeficiency virus type 1 variants. Clin Microbiol Infect 2016; 22:191–200. 10.1016/j.cmi.2015.10.004 26482266

[8] Gilbert M, Ferré VM, Guilbaud R, et al How should APOBEC3-induced resistance mutations be considered in the management of antiretroviral therapy? J Antimicrob Chemother 2025; 80:3133–8. 10.1093/jac/dkaf353 PMC12596043 41016855

[9] Stanford University . Stanford University HIV Drug Resistance Database. APOBEC-associated mutations in HIV drug resistance. Available at: https://hivdb.stanford.edu/page/apobecs/

[10] Pollpeter D, Parsons M, Sobala AE, et al Deep sequencing of HIV-1 reverse transcripts reveals the multifaceted antiviral functions of APOBEC3G. Nat Microbiol 2017; 3:220–33. 10.1038/s41564-017-0063-9 PMC6014619 29158605

[11] Kawale AS, Zou L. Regulation, functional impact, and therapeutic targeting of APOBEC3A in cancer. DNA Repair (Amst) 2024; 141:103734. 10.1016/j.dnarep.2024.103734 PMC11330346 39047499

[12] Jason DS, Ryan PB, H CS. The APOBEC protein family: unit by structure, divergent in function. Trends Biochem Sci 2017; 176:139–48. 10.1016/j.tibs.2016.05.001 PMC4930407 27283515

[13] Ebrahimi D, Alinejad-Rokny H, Davenport MP. Insights into the Motif preference of APOBEC3 enzymes. PLoS One 2014; 9:e87679. 10.1371/journal.pone.0087679 PMC3909203 24498164

[14] Anwar F, Davenport MP, Ebrahimi D. Footprint of APOBEC3 on the genome of human retroelements. J Virol 2013; 87:8195–204. 10.1128/JVI.00298-13 PMC3700199 23698293

[15] Holtz CM, Sadler HA, Mansky LM. APOBEC3G cytosine deamination hotspots are defined by both sequence context and single-stranded DNA secondary structure. Nucleic Acids Res 2013; 41:6139–48. 10.1093/nar/gkt246 PMC3695494 23620282

[16] Xu WK, Byun H, Dudley JP. The role of APOBECs in viral replication. Microorganisms 2020; 8:1899. 10.3390/microorganisms8121899 PMC7760323 33266042

[17] Kim E-Y, Lorenzo-Redondo R, Little SJ, et al Human APOBEC3 induced mutation of human immunodeficiency virus type-1 contributes to adaptation and evolution in natural infection. PLoS Pathog 2014; 10:e1004281. 10.1371/journal.ppat.1004281 PMC4117599 25080100

[18] Caro-Murillo AM, Castilla J, Pérez-Hoyos S, et al Cohorte RIS de pacientes con infección por VIH sin tratamiento antirretroviral previo (CoRIS): metodología y primeros resultados. Enferm Infecc Microbiol Clin 2007; 25:23–31. 10.1157/13096749 17261243

[19] Sobrino-Vegas P, Gutiérrez F, Berenguer J, et al La cohorte de la red española de investigación en sida y su biobanco: organización, principales resultados y pérdidas al seguimiento. Enferm Infecc Microbiol Clin 2011; 29:645–53. 10.1016/j.eimc.2011.06.002 21820763

[20] ABL SA . DEEPchek® software (version 2.0). Available at: https://www.ablsa.com/laboratory-solutions/deepchek-software/

[21] Gifford RJ . Sequence editing by apolipoprotein B RNA-editing catalytic component-B and epidemiological surveillance of transmitted HIV-1 drug resistance. AIDS 2008; 22:2225. 10.1097/QAD.0b013e3282f5e07a PMC2946849 18356601

[22] Rhee S-Y, Sankaran K, Varghese V, et al HIV-1 protease, reverse transcriptase, and integrase variation. J Virol 2016; 90:6058–70. 10.1128/JVI.00495-16 PMC4907232 27099321

[23] Armenia D, Carioti L, Micheli V, et al Comparison of different HIV-1 resistance interpretation tools for next-generation sequencing in Italy. Viruses 2024; 16:1422. 10.3390/v16091422 PMC11437420 39339898

[24] Capina R, Li K, Kearney L, Vandamme AM, Harrigan PR, Van Laethem K. Quality control of next-generation sequencing-based HIV-1 drug resistance data in clinical laboratory information systems framework. Viruses 2020; 12:1–16. 10.3390/v12060645 PMC7354600 32545906

[25] Ng T T-L, Su J, Lao H-Y, et al Long-read sequencing with hierarchical clustering for antiretroviral resistance profiling of mixed human immunodeficiency virus quasispecies. Clin Chem 2023; 69:1174–85. 10.1093/clinchem/hvad108 37537871

[26] Boltz VF, Shao W, Bale MJ, et al Linked dual-class HIV resistance mutations are associated with treatment failure. JCI Insight 2019; 4:1–11. 10.1172/jci.insight.130118 PMC6795402 31487271

[27] Alidjinou EK, Coulon P, Tetart M, et al Defective HIV-1 DNA pol sequences are not associated with HIV-1 DNA levels and drive most APOBEC-context drug resistance mutations. J Antimicrob Chemother 2025; 80:941–6. 10.1093/jac/dkaf016 39849847

[28] Rose PP, Korber BT. Detecting hypermutations in viral sequences with an emphasis on G → A hypermutation. Bioinformatics 2000; 16:400–1. 10.1093/bioinformatics/16.4.400 10869039

